# The Reduction of Salt in Different Cheese Categories: Recent Advances and Future Challenges

**DOI:** 10.3389/fnut.2022.859694

**Published:** 2022-04-04

**Authors:** Flavio Tidona, Miriam Zago, Domenico Carminati, Giorgio Giraffa

**Affiliations:** Council for Agricultural Research and Economics, Research Centre for Animal Production and Aquaculture (CREA-ZA), Lodi, Italy

**Keywords:** salt intake, sodium reduction, cheese ripening, cheese type, sodium chloride replacement

## Abstract

Public awareness about excessive sodium intake and nutrition claims related to salt content entail the need for food industries to carefully reconsider the composition and processing of high sodium foods. Although in some products the reformulation with alternative ingredients is commonly practiced, in cheese the reduction of salt is still a challenging task, as sodium chloride exerts multiple and fundamental functions. Salt favors the drainage of the residual whey, enhances the taste and the aroma profile, regulates the texture, the final pH, the water activity, and affects the microbial growth. Ultimately, salt content modulates the activity of starter and non-starter lactic acid bacteria (NSLAB) during cheese manufacturing and ripening, influencing the shelf-life. Any modification of the salting procedure, either by reducing the level of sodium chloride content or by replacing it with other salting agents, may affect the delicate equilibrium within the above-mentioned parameters, leading to changes in cheese quality. The decrease of Na content may be differently approached according to cheese type and technology (e.g., soft, semi-hard, hard, and mold-ripened cheeses). Accordingly, targeted strategies could be put in place to maintain the overall quality and safety of different cheeses categories.

## Introduction

The relationship between sodium (Na) intake and its immediate effect on blood pressure was already fully demonstrated (1), although several concomitant conditions (hypertension, obesity, age, lifestyle etc.) may predispose or directly cause the onset of cardiovascular diseases. In addition, health impacts related to prolonged intake of excessive salt were postulated (2, 3). To clarify the controversial results obtained so far, clinical trials are being carried out to shed light on the cause-effect linkage between salt assumption and consumer health consequences (4). Health organizations, driven by a greater awareness of public opinion and consumer associations, converge on a unanimous identification of strategies for reducing salt in food as a preventive measure, that might integrate similar initiatives on saturated fats and sugar consumption. In this view, food industries have been considering to carefully revise receipts and process technologies to lower Na content in food. This recommendation can be voluntary applied, but those products that successfully reduce Na content (by 25 or 30% at least) may boast a nutritional claim on the label, an added value compared to analogous products. The reduction of Na in cheeses is difficult to accomplish since salt exerts multiple important roles, contributing to product quality and safety (5). Differently from other foods, where a simple reformulation of the ingredients can be sufficient, a decrease of salt content in cheese would increase the moisture content, the water activity (a_*w*_), the rate of lactose metabolism, and the intensity of proteolytic phenomena. Not less important, a lower rate of bacterial cell autolysis that could interfere on the regular cheese aging could take place (6). As a result, cheeses with reduced salt content may show sensorial defects, modified rheological properties or be more subjected to spoilage or structural problems during shelf-life. Since several cheese typologies can be produced, each showing very different physico-chemical and microbiological characteristics, the reduction of salt may exert product-specific downsides among those previously cited. At the same time, different strategies may be applied to reduce Na content in cheeses according to the specific typology. Some attempts on Na reduction in soft, semi-hard, hard and mold-ripened cheeses are briefly overviewed.

## Soft Cheeses

Cheeses are perceived as a Na-rich food category, even if soft cheeses may contain wide range (0.7–2.3%) of NaCl, with different related impacts (7, 8). Salt is one of the main obstacles to control the development of bacteria in cheese. Soft cheeses are generally characterized by relatively low fat and high moisture content; thus, the reduction of salt may lower the salt-in-moisture (S/M) ratio, which implies possible adverse effects on the safety and shelf life of the products (9). The simplest approach to reduce Na in soft cheeses is to decrease the rate of NaCl addition or to use other chloride salts, such as KCl, CaCl_2_ or MgCl_2_ (10). Salt reduction or the use of salt replacers must be carefully evaluated since the physico-chemical characteristics of soft cheeses allow the multiplication of pathogenic and spoilage bacteria. Specifically, a suitable selection of the LAB starter culture is fundamental to ensure the safety of these cheese through the rapid production of organic acids (mainly lactic acid) and other products derived from the secondary bacterial metabolism (i.e., hydrogen peroxide, enzymes or bacteriocins), thus exerting a competitive effect against undesired microorganisms (11, 12). Besides, the replacement of NaCl with KCl may have positive effects as the 50% substitution of the brine solution, with reduced concentration (7.5%), enhanced the survival of added probiotic bacteria and significantly increased the release of 4 essential amino acids, i.e., phenylalanine, tryptophan, valine, and leucine (13).

To inhibit microbial spoilage, often detected during the shelf-life of perishable soft cheeses with reduced Na content, a natural additive was tested to prevent the growth of molds; to this end, *Nigella sativa* oil was added to pasteurized milk before renneting and showed antifungal properties against *Candida albicans* and *Aspergillus parasiticus* after 14 days of storage of a soft, low-salt cheese (14).

The concentration of ions such as Na^+^ and K^+^ also affects the activity of enzymes, although in mozzarella with low Na, the level of proteolysis did not change significantly, probably because of its short shelf-life (15). Similar findings were reported when NaCl was substituted with KCl in other soft cheeses such as Halloumi, Feta and white cheeses (16–18). Based on sensory properties, research on mozzarella and cottage cheese produced with 30–35% less NaCl did not highlight differences in the overall acceptability; however, higher rates of reduction negatively impacted on sensorial traits (19).

An alternative approach to reduce Na content in white cheese was carried out preparing brine solution with 8 and 12% of hydrocolloids, showing promising results with carrageenan and gelatin rather than guar or xantan gum which negatively influenced flavor, texture and appearance (20).

## Semi-Hard and Hard Cheeses

Often, long ripened cheeses include typical and PDO (Protected Designation of Origin) cheeses that may be produced from raw milk. Although in long ripened cheeses salt is added when the curd is already structured, post-production contamination can occur, especially during some delicate steps, e.g., cheese shredding or grating. In a study conducted by Hystead et al. (21) a pool of *Listeria monocytogenes* strains was inoculated in Cheddar cheese salted with K and low Na at different ages: no differences in the survival of the pathogen were observed, except for the 33rd week of age when survival of *L. monocytogenes* was significantly higher in the Na-reduced cheeses. This was related to the increase of pH due to a more intense proteolysis and the subsequent higher availability of free amino acids that could favor the stress adaptation abilities of the pathogen. The replacement of Na with K in Edam cheese did not affect the bacterial composition of the reformulated cheese when intentionally contaminated by *Listeria innocua* (22). In low-salt Cheddar, a survival of *Salmonella* spp. was detected up to 90 days when the cheese was stored at 4 or 10°C and for up to 30 days at 21°C (23). Several attempts to reduce NaCl content in Cheddar cheese were carried out, highlighting contrasting and non-definitive results. The employment of high rate of NaCl substitution (> 30%) with MgCl_2_, KCl, or CaCl_2_ induced defects in terms of texture, onset of metallic flavor, and a decay of the sensory properties, especially due to undesirable aftertaste (11, 24, 25). Reduction of salt up to a certain extent in hard cheeses often led to higher microbial growth and proteolysis, affecting hardness and gumminess of resulting cheeses (26). Nevertheless, low Na Cheddar cheeses with overall good quality were achieved in other studies (19, 27). Since the delicate equilibrium that salt exerts on cheeses, some processing conditions in Cheddar-type cheeses could be optimized (e.g., reducing residual lactose in the curds, rennet type dosage, addition of starter/adjunct cultures, or use of ultrafiltered retentate to milk) to counterbalance the negative effects of salt replacement or reduction (24). The investigation on physico-chemical and sensory properties of Cheddar cheese with different salt contents showed a gradual deterioration of flavor upon a 50% salt reduction. However, moisture regulation in cheesemaking, mainly obtained by reducing the curd grain cutting size and extending the time of cooking at 40°C, proved to be the most effective technological operation that allowed to deliver Cheddar cheeses with restored textural properties (27). In a Grana-type hard cheese, the substitution of NaCl with KCl at 30 or 50% enabled to produce comparable cheeses with no significative differences in terms of chemical composition, microbial counts, and sensory evaluations (28).

## Mold-Ripened Cheeses

In mold-ripened cheeses such as Stilton, Camembert and Gorgonzola, the selection of the fungal strains employed is of paramount relevance to enable a regular growth, which consequently affects the development of the bloomy aspect and the typical sensory properties due to lipolysis and proteolysis. Mold-ripened cheeses include two different typologies based on the inoculum distribution of the molds added as secondary starter: (i) surface-ripened cheeses, when the inoculum is spread onto the rind before ripening and (ii) blue-veined cheeses, when spores of specific mold species are directly added to milk in vat before cheesemaking. The salt levels in surface-ripened cheeses may vary from 1.5 to 2.3%, although at the beginning of ripening salt is still more concentrated in the rind, thus allowing highly adapted microbial species to grow at low pH and high salt content. This is particularly evident in washed-rind cheeses such as Taleggio, Gruyere, Epoisses, Tilsit, and Limburger where a wide microbial community, including molds, spontaneously colonizes the cheese surface (29, 30). The main microbial genera detected in the rind belong to halotolerant microorganisms (e.g., *Penicillium, Geotrichum*, *Debaryomyces*, *Brevibacterium*, *Corynebacterium*, and *Microbacterium*). The wide microbial community positively impact on the quality and organoleptic characteristics of ripened cheeses and also acts as a physical and biological barrier against pathogens and spoilage microorganisms (30). The reduction of salt in surface-ripened cheeses may lead to spoilage problems, probably as a consequence of modified equilibria on the halophilic microbial community. For example, the growth of *P. camemberti* in Camembert cheese salted with 0.8% of NaCl resulted poor and irregular (11). Dugat-Bony et al. (8) showed significantly higher growth of an intentionally inoculated microorganism (*Pseudomonas fragi*) on a surface-ripened cheeses with a lower concentration of salt (1.3%) compared to control (1.8%). The production of Camembert cheese with a 20% of NaCl reduction, partially substituted with KCl, did not affect the physico-chemical properties, the microbial counts of the adjunct starter culture (*Geotrichum candidum* and *P. camemberti*, 1:2) and the spoilage bacteria, as well as the sensory acceptance (31). RNA-seq analysis highlighted a relative increase of *G. candidum* in the NaCl-reduced Camembert cheese, leading to an unbalanced growth of the molds. Even if not investigated in that study, lowering the salt content may allow *G. candidum* to outcompete *P. camemberti*, resulting in cheeses with a higher ammoniacal flavor and bitterness, given the strong proteolytic activity associated to *G. candidum* (31), or leading to a surface defect called “toad skin” (32).

Blue-veined cheeses are among the most heavily salted varieties, with NaCl concentrations ranging from 3 to 5%, often salted with dry salt. The maturation process of mold-ripened cheeses largely depends on the growth of the inoculated spores of *Penicillium roqueforti* whose germination is stimulated by the presence of at least 1% of NaCl. To this regard, the influence of salt on lipolysis was investigated, displaying a dose-dependent effect: no statistical differences in lipolysis were reported when the content of NaCl fell in the range of 0.5–3%, while intensifying the level of NaCl to around 4–6% an increased lipolysis can occur; at higher salt concentrations lipolysis tended to decrease (10).

## Discussion

Up to now, although lack of knowledge still exists, several reports suggests that Na directly damages target organs via multiple intricate pathways, therefore further efforts should be done to reduce Na in cheeses as feasible strategy for maintaining the human health state (33). The selection of suitable starter cultures could be a strategic key to compensate salting modifications, either by ensuring safety requirements and contributing to improve the nutritional value and the sensory properties. In low salt cheeses, the use of coagulants alternative to chymosin, such as from camel chymosin, and specific lactic acid bacteria can offer the opportunity to limit the production of bitter peptides and accumulate flavor enhancing compounds (34). The modifications induced by this practice, combined with salt reduction, may also influence the overall microbial metabolism, favoring the release of compounds with health concern (e.g., biogenic amines from enterococci) in cheese (35). For instance, the level of putrescine and cadaverine was found at higher concentrations in a soft surface-ripened cheese with reduced salt content (8). At the same time, the enhanced development of either the starter culture or the non-starter lactic acid bacteria (NSLAB) in salt reduced, or differently salted, cheeses may lead to the accumulation of benzoic acid, mainly originated from the microbial conversion of hippuric acid (36). Benzoic acid is a common antimicrobial preservative that cannot be added to dairy products, but it can naturally occur at variable amounts according to milk species, starter composition, and fermentation conditions (37). Therefore, the effect of different salting procedures on the unpredicted formation of such secondary metabolites should be worthy of investigation.

**FIGURE 1:**
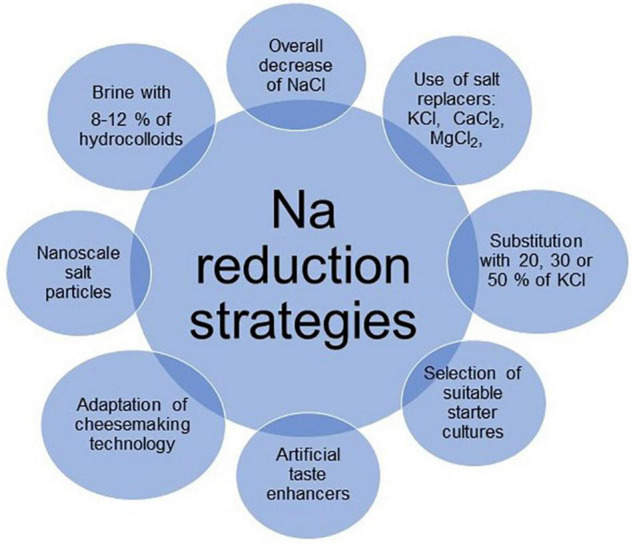
Some strategies investigated to reduce Na content in cheeses.

For cheeses with a limited ripening and perishable characteristics, the reduction of salt needs to put in place other hurdles during processing, principally good hygienic practices and low temperature (4°C) soon after manufacture, to avoid product deterioration during shelf life. In hard and mold-ripened cheeses, Na reduction or substitution needs to be case by case investigated to ensure a regular ripening process, related to proteolysis and lipolysis, together with sensorial acceptance. Among the possible strategies investigated (Figure 1) the employment of KCl for several types of cheeses appeared one of the most successful applications to reduce Na content with limited drawbacks. Moreover, K intake may have a positive effect on human health on the state of kidneys, heart, muscles, and the chemical transmission through the nervous system (38). The use of artificial taste enhancers such as monosodium glutamate, yeast extracts, hydrolyzed vegetable proteins or disodium inosinate may offer an increased salty perception, but they still have a negative impact on consumer acceptance when listed on the label (24, 39). Additionally, reducing the particle size of salt by nanoscale spray-drying method increased saltiness perception allowing a reduction of Na from 25 to 50% in surface-salted cheese crackers without adverse influence on sensory attributes (40). Potential benefits could therefore arise from the application of such nanotechnology to the dairy industry. The future research focused on Na reduction in cheeses still may explore different approaches, but it should take into account multiple elements, recent scientific advancements and even marketing and consumer trends.

## Author Contributions

FT was responsible for main manuscript writing. GG provided full revision. DC searched updated bibliography and revision. MZ edited the final version. All authors contributed to the article and approved the submitted version.

## Conflict of Interest

The authors declare that the research was conducted in the absence of any commercial or financial relationships that could be construed as a potential conflict of interest.

## Publisher’s Note

All claims expressed in this article are solely those of the authors and do not necessarily represent those of their affiliated organizations, or those of the publisher, the editors and the reviewers. Any product that may be evaluated in this article, or claim that may be made by its manufacturer, is not guaranteed or endorsed by the publisher.
